# Supplementary material for: The Systems Biology Research Tool: evolvable open-source software
Source: BMC Syst Biol. 2008 Jun 29;2:55. doi: 10.1186/1752-0509-2-55 (PMC2446383; doi:10.1186/1752-0509-2-55)
Supplement: Additional file 1 — SBRT Archive. An archive of the current version of the Systems Biology Research Tool. [file 1752-0509-2-55-S1.zip › sbrt-1.4.0/doc/developers_guide/api/sbrt/kernel/math/graph_theory/package-use.html]

Uses of Package sbrt.kernel.math.graph\_theory


|  |  |  |
| --- | --- | --- |
| **Overview** \| **Package** \| Class \| **Use** \| **Tree** \| **Deprecated** \| **Index** \| **Help** | |  |
| PREV   NEXT | **FRAMES**    **NO FRAMES**     **All Classes** |  |


---


## **Uses of Package sbrt.kernel.math.graph\_theory**

| Packages that use sbrt.kernel.math.graph\_theory | |
| --- | --- |
| **sbrt.kernel.math.graph\_theory** | Provides classes and interfaces for creating objects relevant to graph theory. |
| **sbrt.kernel.math.graph\_theory.comp** | Provides classes and interfaces for performing graph theoretical computations. |
| **sbrt.kernel.math.graph\_theory.proc** | Provides classes and interfaces for performing graph theoretical processes. |
| **sbrt.shell.mng.graph\_theory** | Provides classes and for creating graph theoretical process managers. |
| **sbrt.shell.text.math** | Provides classes and interfaces for defining formats for mathematical objects. |

Classes in sbrt.kernel.math.graph\_theory used by sbrt.kernel.math.graph\_theory

****DirectedGraph.Edge****
  
          This class is used to represent the unidirectional
edges of a directed graph.

Classes in sbrt.kernel.math.graph\_theory used by sbrt.kernel.math.graph\_theory.comp

****DirectedGraph****
  
          This class is used to represent directed graphs.

****Path****
  
          
This class can be used for representing paths in a graph
or network.

Classes in sbrt.kernel.math.graph\_theory used by sbrt.kernel.math.graph\_theory.proc

****DirectedGraph****
  
          This class is used to represent directed graphs.

****Path****
  
          
This class can be used for representing paths in a graph
or network.

Classes in sbrt.kernel.math.graph\_theory used by sbrt.shell.mng.graph\_theory

****DirectedGraph****
  
          This class is used to represent directed graphs.

****DirectedGraph.Edge****
  
          This class is used to represent the unidirectional
edges of a directed graph.

Classes in sbrt.kernel.math.graph\_theory used by sbrt.shell.text.math

****DirectedGraph.Edge****
  
          This class is used to represent the unidirectional
edges of a directed graph.

---


|  |  |  |
| --- | --- | --- |
| **Overview** \| **Package** \| Class \| **Use** \| **Tree** \| **Deprecated** \| **Index** \| **Help** | |  |
| PREV   NEXT | **FRAMES**    **NO FRAMES**     **All Classes** |  |


---
